# Young people's compliance with the Experience Sampling Method (ESM): Examining patterns, predictors and associations with well-being and mental health

**DOI:** 10.1016/j.invent.2025.100859

**Published:** 2025-07-08

**Authors:** Julius März, Lianne P. de Vries, Hanneke Scholten, Annabel Vreeker, Jeroen S. Legerstee, Loes Keijsers, Brenda W.J.H. Penninx, Manon H.J. Hillegers

**Affiliations:** a Department of Child and Adolescents Psychiatry/Psychology Erasmus MC Sophia Children's Hospital, Erasmus University Medical Center, Rotterdam, the Netherlands; b Department of Biological Psychology, Vrije Universiteit Amsterdam, Amsterdam, the Netherlands; c Department of Psychology, Education & Child Studies, Team Youth and Family, Erasmus School of Social and Behavioural Sciences, Erasmus University Rotterdam, the Netherlands; d Department of Psychiatry, Amsterdam UMC, Vrije Universiteit Amsterdam, Amsterdam, the Netherlands

**Keywords:** Experience sampling method, Mobile mental health, Compliance, Young people, Well-being, Latent class growth analysis

## Abstract

The Experience Sampling Method (ESM) can help young people gain insight into their fluctuating emotions through multiple daily surveys. This can act as an intervention to improve well-being and mental health. However, the effectiveness of ESM interventions is expected to depend on compliance, i.e., how often participants respond to these surveys. We aimed to understand compliance patterns among young people during an ESM-based intervention, explored predictors of these patterns, and examined if the intervention's impact on well-being and mental health varied with compliance levels.

Dutch adolescents and young adults (*N* = 1139, 12–25 years, mean age = 17.67; 79 % female) completed baseline questionnaires, responded to five daily ESM surveys over three weeks using the Grow It! app, and completed follow-up questionnaires.

Despite overall low compliance (mean compliance = 33.8 %), latent class growth analyses identified four compliance patterns: stable high (*N* = 176; M = 78.8 %), stable medium (*N* = 193; M = 50.1 %), high initial and decreasing (*N* = 272; M = 30.9 %), and low initial and decreasing (*N* = 498; M = 13.2 %). These patterns were not consistently associated with age, gender, education, baseline well-being, or depressive and anxiety symptoms, and did not influence the intervention's effect on well-being and mental health outcomes.

We identified specific ESM compliance patterns among young people but found no clear predictors or outcomes of these patterns. To improve compliance and intervention effectiveness, future ESM interventions can potentially be enhanced by personalized designs, incorporating intrinsic and extrinsic motivators, and investigating situational factors and additional participant characteristics.

## Highlights

• 1139 young people used Grow It!, a three weeks ESM mobile mental health intervention
• We identified four distinct patterns of ESM compliance over time between participants
• ESM compliance patterns were unrelated to demographics and baseline mental health
• Differences in compliance did not affect changes in well-being and mental health
• Well-being and mental health improved in participants across all compliance levels

## 1 Introduction

Worldwide numbers indicate that around 31 % of children under 18 show symptoms of anxiety and depression (Deng et al., 2023). In the Netherlands, over 50 % of youth aged 16 to 25 experience mental health problems (RIVM, 2023). Meanwhile, capacities for in-person mental health services are increasingly overwhelmed (McGorry et al., 2022), with long waiting times for in-person care (Radez et al., 2021). Therefore, additional approaches are needed to support mental health in young people. In recent years, mobile mental health (mHealth) apps have become increasingly popular (Camacho et al., 2022). mHealth apps can support young people with mental health problems, for example by providing Cognitive Behavioral Therapy (CBT) based interventions (Beck and Aaron Beck, 2020; Lehtimaki et al., 2021). More and more mHealth apps implement these digital interventions that can deliver fundamental techniques of CBT like behavioral activation or cognitive restructuring, without having actual therapist-patient interactions (Denecke et al., 2022). In healthy young people, mHealth apps can promote well-being and prevent mental health problems, for example by improving mental health literacy and providing psychoeducation (Martinengo et al., 2022).

The rising popularity of mHealth apps (Camacho et al., 2022) goes along with the increasing use of the Experience Sampling Method (ESM) in clinical research and interventions (van Roekel et al., 2019). With ESM, participants receive multiple surveys throughout the day via their smartphone, asking them about their current emotions (e.g. “How sad are you right now?”), behavior, and (social) context (Csikszentmihalyi and Larson, 1987; Shiffman et al., 2008; van Berkel et al., 2017). This extended and ecologically valid assessment of daily affect and environment can help identify emerging and current mental health problems in young people (Kuranova et al., 2021; Marciano et al., 2023). Next to ESM as an empirical assessment tool of daily affect, the self-monitoring aspect of ESM could potentially serve as an intervention, by increasing a young person's perception of their emotional fluctuations, awareness of positive and negative events, and appreciation for natural rewards, like pleasant activities (van Os et al., 2017). ESM has therefore become an integral part of many mHealth apps (Dietvorst et al., 2024a).

However, the successful implementation of ESM in affect assessment and interventions is expected to depend on compliance, i.e., the number of surveys participants complete. Many studies deal with low compliance (van Roekel et al., 2019), as participants can perceive repeated questionnaires as burdensome (Piot et al., 2022). However, this could undermine the study's quality. Low ESM compliance reduces the statistical power to detect effects in clinical research due to more missing values (Fuller-Tyszkiewicz et al., 2013; Schuster et al., 2020). With ESM as an intervention tool, answering surveys is even more important, as reduced ESM compliance could impair young people's insights into their emotions and limit potential positive effects of self-monitoring on well-being and mental health.

Research into intervention effects of self-monitoring via ESM on emotional self-insight, well-being, and mental health is rare. Van Os et al. (2017) reviewed ESM designs and found self-monitoring to positively affect resilience in adults. Similarly, Widdershoven et al. (2019) found that self-monitoring via ESM improved the differentiation of negative emotions. However, neither study examined the impact of different compliance levels on self-monitoring. Furthermore, the effect of emotional self-monitoring via ESM on well-being and mental health in adolescents is unknown, despite increased emotional awareness being associated with lower anxiety and depression (Sendzik et al., 2017). In this study, we aim to examine patterns of compliance of young people throughout an ESM intervention and assess whether the characteristics of participants affect compliance. Next, we address the concern that low compliance undermines an intervention's effect and examine whether compliance patterns in an ESM intervention are associated with the strength of improvements in well-being and mental health in young people.

### 1.1 Examining ESM compliance in interventions

In a recent review, van Roekel et al. (2019) demonstrated that average ESM compliance varies widely between studies with adolescents (range = 51 % to 92 %). However, a detailed examination of individual compliance differences between participants is needed, as assessing average compliance as the only metric can lead to reductive perceptions of ESM compliance (Stone et al., 2023). For example, an intervention's efficacy might differ strongly between participants with the same average compliance, when one steadily completes 50 % of ESM surveys and the other starts with 100 % compliance and decreases to 0 %. Therefore, researchers suggest also assessing compliance patterns over time (Trull et al., 2020; van Roekel et al., 2019). Studies on ESM compliance trajectories showed compliance on average declining over time across participants (e.g. Eisele et al., 2022; Ono et al., 2019). However, some young people might show a strong decline in compliance, whereas others show stable compliance levels or an increase over time. Therefore, our study's first aim is to explore if different ESM compliance patterns can be identified among young people aged 12–25 years.

### 1.2 Participant characteristics impacting ESM compliance

Our second aim is to examine if these established compliance patterns are related to participant characteristics. A comprehensive meta-analysis found no clear relationship between age, gender, or other participant characteristics and ESM compliance in adolescents (Wen et al., 2017). However, most studies related participant characteristics to average compliance. Meanwhile, linking compliance patterns throughout the intervention to participant characteristics could identify which participants might engage with an ESM intervention consistently, drop out, or show increasing participation. Furthermore, with ESM as an intervention tool, one must account for baseline well-being and mental health (e.g. depressive and anxiety symptoms) of young people, as initial well-being might affect the motivation and ability to engage with ESM surveys.

### 1.3 ESM compliance and the intervention's efficacy

Our third aim is to examine whether different compliance patterns are associated with intervention-related changes in well-being and mental health. ESM can strengthen resilience through emotional self-insight (van Os et al., 2017), but it is unknown if ESM compliance influences changes in well-being and mental health throughout interventions. In a systematic review of mHealth interventions targeting depression, no ESM intervention in adults examined whether higher compliance was associated with stronger clinical improvements (Molloy and Anderson, 2021). However, we hypothesize that higher compliance in an ESM intervention leads to better well-being and mental health outcomes in young people. A comprehensive examination of these associations can support the clinical evaluation of ESM interventions.

### 1.4 The current study

In this preregistered study (https://osf.io/xu23s/?view_only=a8dd3da5038e4c20b1769afc1676a9ea) we aimed to better understand young people's ESM compliance and its relationship to participant characteristics and a potential intervention effect on well-being and mental health. We used data from “Grow It!”, a multiplayer serious gaming app designed to improve self-insight and adaptive coping in young people aged 12 to 25 years (Dietvorst et al., 2022). Users of Grow It! received five ESM surveys per day over three weeks. Additionally, participants received daily Cognitive Behavioral Therapy (CBT) based challenges designed to improve coping strategies. Participant characteristics, including age, gender, education status, well-being, depressive symptoms, and anxiety symptoms were assessed before and after using the Grow It! app. We have three study aims:

1. We investigate whether there are distinct patterns of ESM compliance among young people. We expect at least two classes of participants with distinguishable patterns of compliance to ESM surveys, i.e., stable high compliance, and stable low compliance (H1).
2. We explore the relationship between baseline participant characteristics of young people (i.e., age, gender, education, cognitive and affective well-being, and symptoms of depression and anxiety) and ESM compliance patterns.
3. We examine whether compliance patterns (e.g., stable high compliance and stable low compliance) relate to changes in cognitive and affective well-being and symptoms of depression and anxiety throughout the intervention. We hypothesize (H2) that higher compliance classes show stronger increases in cognitive and affective well-being throughout the intervention than lower compliance classes. Furthermore, we explore if young people in higher compliance classes show stronger decreases in depressive and anxiety symptoms.

## 2 Methods

### 2.1 Participants

Dutch youth between 12 and 25 years participated in a prospective longitudinal online study in two consecutive cohorts during the COVID-19 pandemic: cohort 1 from December 2020 to March 2021 and cohort 2 from March 2021 to May 2021 (see Grow It! Corona project: https://osf.io/2at58/?view_only=b691104ecc3d45ad8b48e1bd60ad7125). It was approved by the METC of the Erasmus Medical Center [MEC-2020-0287]. Only participants in cohort 2 received personalized mood profiles, which provided each participant with visual representations of their personal mood, based on their answers in the ESM surveys (see 2.4.). However, this mood profile did not significantly improve compliance or influence the intervention's effect on well-being and mental health (Dietvorst et al., 2024b). Therefore, we treated them as a singular cohort. In total, *N* = 1139 participants completed baseline questionnaires and at least 5 % of ESM surveys, while *N* = 758 participants completed follow-up questionnaires too (see Table 1). *N* = 886 participants were excluded for completing <5 % of ESM surveys. Included participants were on average 17.67 years old (*SD* = 3.44; range: 11.7–25.5), the majority were female (79 %) and of Dutch ethnicity (92 %). On average, included participants showed low baseline scores of depressive symptoms and anxiety and moderately high scores of affective and cognitive well-being (see Table 1; also Dietvorst et al. (2022) and Dietvorst et al. (2024b)).

**Table 1:** Sample characteristics and descriptive statistics.

	Baseline (*N* = 1139) M (SD) or %	Follow-up (*N* = 758) M (SD) or %
Demographic variables		
Age	17.67 (3.44)	17.70 (3.38)
Gender (% female)	79 %	80 %
Ethnicity	92 % Dutch	93 % Dutch
	5 % Mixed	5 % Mixed
	3 % Other	2 % Other
Education level	1 % Primary	1 % Primary
	16%Low	14 % Low
	29 % Middle	29 % Middle
	54 % High	56 % High

Study outcomes		
ESM Compliance %	33.8 % (24.50)	41.3 % (25.25)
Affective Well-being	4.47 (1.39)	4.78 (1.37)
Cognitive Well-being	5.82 (2.24)	6.24 (2.20)
Depressive symptoms	6.98 (4.52)	6.62 (4.51)
Anxiety symptoms	8.76 (4.65)	8.47 (4.66)
Covid stringency	76.92 (4.46)	77.28 (4.19)

Note. Education level refers to primary school (primary), preparatory school for technical and vocational training (low), preparatory school for higher vocational education (middle), and preparatory school for university (high). COVID stringency refers to averaged scores of the daily changing index of COVID containment efforts in the Netherlands.

### 2.2 Procedure

Online recruitment took place through schools and (social) media. If registered via the study website (https://growitapp.nl), young people and - for participants younger than 16 - their parents signed informed consent via a secure webpage and filled out baseline online questionnaires. Subsequently, they received a unique activation code for the Grow It! app per SMS, activating it for three weeks. Participants received five ESM surveys per day, with 105 surveys in total. They also received three different challenges every day (e.g., “Take a picture of a red car!” or “Write down what a friend of yours likes about you!”) and could complete one. Challenges were based on Cognitive Behavioral Therapy (CBT) to support adaptive coping (Connor-Smith et al., 2000; Legerstee et al., 2010). With ESM and challenges encompassing two separate mechanisms for improving well-being, we focused on the ESM part of the intervention. After the three-week ESM period, follow-up questionnaires were completed.

### 2.3 Variables

#### 2.3.1 Sociodemographics

Baseline items measured gender (male/female/other/don't want to say), age, and ethnicity (Dutch/Non-Dutch/Mixed). Current educational level was measured with four levels, ranging from primary school (coded as 0) to lower education (1: preparatory school for technical and vocational training), intermediate/middle education (2: preparatory school for higher vocational education) and higher education (3: preparatory school for university).

#### 2.3.2 Well-being and mental health

Mental health characteristics and well-being were assessed at baseline and follow-up.

Affective well-being was assessed with one item (“How happy did you feel in the past week on a scale from 1 (Not at all) to 7 (Totally)?”). Cognitive well-being was assessed with one item (“How satisfied were you with your life in the past week on a scale from 1 (Not at all) to 10 (Totally)?”; Tabor and Yull, 2018). These single-item measurements of affective and cognitive well-being have shown good convergent validity and comparable reliability to multi-item scales in previous research (e.g. strong correlations between affective well-being item and affect scales in Beyens et al., 2020; strong correlations between cognitive well-being item and abbreviated life satisfaction scales in Cheung and Lucas, 2014).

Depressive symptoms were measured with the Children's depression inventory II (CDI-II) (baseline & follow-up α = 0.85; Kovacs and Preiss, 1992, Timbremont et al., 2008). Items asked participants to select one of three statements best describing their feelings over the past week (e.g., “I am sad sometimes” vs. “I am often sad” vs. “I am sad all the time”). Items were coded on a three-point scale from “0 (low)” to “2 (high)”. A higher combined score of all items indicated stronger depressive symptoms.

Anxiety symptoms were measured by the general anxiety disorder subscale of the Screen for Child Anxiety-related Disorders (SCARED) questionnaire (baseline & follow-up α = 0.87; Birmaher et al., 1997), with items asking participants to describe their feelings over the past 2 weeks (e.g., “In the last 2 weeks I was nervous”). Items were coded on a scale from 1 (Not at all) to 3 (Often/Clearly). A higher combined score of all items indicated stronger anxiety symptoms.

#### 2.3.3 COVID-19 stringency

For each participant, we calculated the average COVID-19 stringency index (Mathieu et al., 2020), based on participation dates and the corresponding stringency of COVID-19 containment efforts (e.g. lockdown) in the Netherlands, to account for potential effects of the COVID-19 pandemic on ESM compliance, mental health, and well-being.

### 2.4 ESM intervention

Users of the Grow It! app received five ESM surveys per day for three weeks, asking them about their positive affect (“How relaxed/happy/satisfied/confident are you right now?“), negative affect (‘How angry/nervous/annoyed/sad are you right now?’) and other mood-related states (”How lonely/tired/bored/worried are you right now?”). In addition, participants indicated where they were and with whom. In the morning survey, they also reported about their previous night's sleep quality. In the evening survey, they were asked additional questions about their physical activity, the most positive and negative events that day, coping strategies with stressful events, the atmosphere at home, and COVID-related worries (see https://osf.io/2at58/?view_only=b691104ecc3d45ad8b48e1bd60ad7125). This completion of questions about mood, whereabouts, and events is expected to increase self-awareness. To strengthen this, participants in cohort 2 received a mood profile, to self-examine their reported mood at different time points of the intervention (see Dietvorst et al., 2024b).

### 2.5 Statistical analysis

The analyses were preregistered at https://osf.io/xu23s/?view_only=a8dd3da5038e4c20b1769afc1676a9ea. All data were analysed using R Version 4.3.1 (R Core Team, 2023). In addition to the preregistered plan, a non-significant Little's MCAR test (Little, 1988) suggested that data were missing completely at random (*X*^*2*^(1, *N* = 1139) = 1.29, *p* = .25). To account for multiple testing, we applied false discovery rate (FDR; Storey, 2010) to determine the threshold of statistical significance for each *p*-value. We calculated separate FDR values for each analysis.

#### 2.5.1 Compliance trajectories of participants

We used latent class growth analysis (LCGA; Jung and Wickrama, 2008) to identify different patterns of young people's ESM compliance, using the ‘lcmm’ package (Proust-Lima et al., 2023). Only participants who completed at least 5 % of surveys were included, to avoid a disproportionately large class of participants without any compliance (see also Dietvorst et al., 2023). A survey was coded as 1 if completed within 45 min after the prompt and 0 if it was not completed. This resulted in 105 (21 days * 5 surveys per day) coded time points. We fit latent growth models to all time points to identify longitudinal compliance patterns. Subsequently, models with different numbers of compliance classes were compared on the Akaike information criterion (AIC), and Bayesian information criterion (BIC) with lower values indicating a better model fit. Furthermore, posterior probabilities, entropy, and the substantive interpretability of the different classes were considered during model selection. No maximum number of classes was specified beforehand. Keeping in mind sample size requirements for stable and trustworthy parameters in subsequent analyses (Hox and McNeish, 2020), a class was only identified if it included at least 50 participants.

#### 2.5.2 Participant predictors of adolescent compliance

We used multinomial logistic regressions to test associations between participant characteristics and compliance classes, using the ‘nnet’ package (Venables and Ripley, 2002). We conducted separate regression models with compliance class as outcome variable and age, gender, education status, and baseline levels of either affective well-being, cognitive well-being, depression, or anxiety as predictors. With only 7 participants identifying as “other gender”, leading to unreliable regression parameters, gender was entered as binary predictor (female vs non-female). COVID-19 stringency was added as covariate. The compliance classes were compared to each other, with the class with the most participants as reference group. For a model including additionally measured baseline variables (loneliness, maladaptive and adaptive coping, psychological treatment) see supplementary Tables S1 and S2.

#### 2.5.3 Association between compliance classes and changes in well-being and mental health

To identify the impact of ESM compliance on changes in well-being and mental health due to Grow It!, we fit separate multilevel models for the different compliance patterns, using the ‘lmerTest’ package (Kuznetsova et al., 2017). For each compliance class, we compared means and confidence intervals of affective well-being, cognitive well-being, and depressive and anxiety symptoms, at baseline and follow-up (T1 and T2). We also performed paired comparisons between all compliance classes at T1 and T2 in a mixed ANOVA. The two time points were nested within participants, with the participant's age, gender, education, and COVID-19 stringency added as covariates.

### 2.6 Deviations from the preregistration

There are some deviations from our preregistration (see at: https://osf.io/xu23s/?view_only=a8dd3da5038e4c20b1769afc1676a9ea). We had planned to compare trajectories of positive and negative affect between compliance classes. However, because overall compliance was very low (*M* = 33.8 %), we could not reliably compare the development of daily positive and negative affect between classes. Due to the missing data, we also could not reliably assess predictors of momentary compliance after all.

When replicating the analyses with young people who used Grow It! for six weeks (*N* = 496) we found similar results. While we only identified three compliance classes, other results were similar to the three-week cohort (see supplementary Tables S7 to S13).

## 3 Results

### 3.1 Demographics

There were no significant correlations between average compliance levels and demographics, well-being, and mental health variables. However, the well-being and mental health variables were strongly correlated with each other (see Table 2).

**Table 2:** Correlations between baseline predictors and compliance.

Variable	1	2	3	4	5	6	7
1. Compliance (%)	–						
2. Age	0.07	–					
3. Education	0.08	−0.07	–				
4. Affective Well-being	−0.04	−0.30	0.13	–			
5. Cognitive Well-being	−0.02	−0.35	0.13	**0.81**	–		
6. Depression	−0.03	0.24	−0.22	**−0.68**	**−0.70**	–	
7. Anxiety	−0.02	0.33	−0.12	**−0.57**	**−0.58**	**0.73**	–

*Note.* Bold font indicates significance after applying FDR correction.

### 3.2 Compliance trajectories of participants

**Fig. 1:**
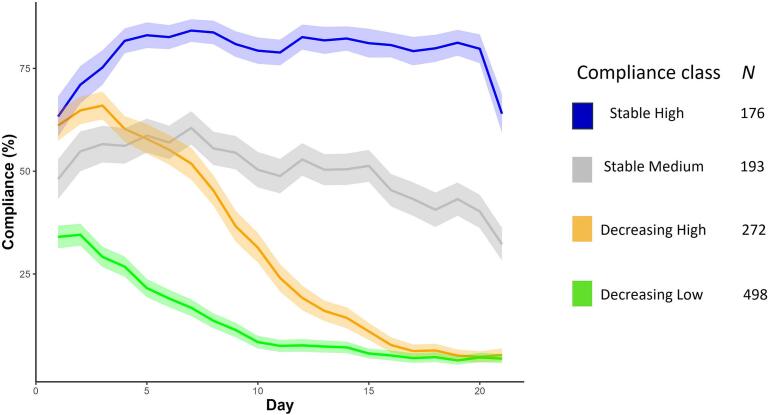
Trajectories of different compliance classes. *Note.* Lines represent mean compliance levels per day. Shades represent 95 % confidence intervals.

Our first aim was to identify different patterns of ESM compliance over three weeks among young people who used the Grow It! app. Latent class growth analyses identified four different ESM compliance classes (see Fig. 1). This 4-class model showed lower AIC and BIC levels than a 3-class solution while showing similarly high posterior probabilities for class membership (>95 % per class; see supplementary Table S3). A 5-class model only showed a slight reduction in AIC and BIC, while showing identical levels of entropy and posterior probabilities as the 4-class solution. However, the fifth class only contained 7.6 % of participants without adding substantive interpretative value, so the 4-class model was selected. The entropy of this solution was 0.96, indicating excellent class separation.

We identified a class of participants that consistently showed high compliance (*mean overall* compliance = 78.8 %; *mean* compliance first day = 63.2 %; labeled “Stable High”), a class of participants that showed continuous medium compliance (*mean overall* compliance = 50.2 %; *mean* compliance first day = 48.1 %; labeled “Stable Medium”), a class of participants that started with high compliance levels that strongly decreased over time (*mean overall* compliance = 30.9 %; *mean* compliance first day = 61.1 %; labeled “Decreasing High”) and a class of participants that started with low compliance that decreased over time (*mean overall* compliance = 13.2 %; *mean* compliance first day = 34.1 %; labeled “Decreasing Low”, see Fig. 1). Sample size, age, and gender distributions of each compliance class are shown in Table 3.

**Table 3:** Sample distribution by compliance class.

Compliance class	*N*	Intercept (SD)	Slope	*M Compliance*	*M* Age	Gender (%Female)
Stable High	176	0.77 (0.005)	0.0003	78.8 %	18.2	82.9 %
Stable Medium	193	0.59 (0.008)	−0.002	50.1 %	18.3	81.3 %
Decreasing High	272	0.69 (0.007)	−0.007	30.9 %	17.0	78.3 %
Decreasing Low	498	0.29 (0.004)	−0.003	13.2 %	17.5	77.7 %

### 3.3 Participant predictors of adolescent compliance

The second aim was to assess the relationship between the four established compliance patterns and participant characteristics. Four different compliance classes were compared in multinomial regressions, with the “decreasing low” compliance class as reference group.

Older participants were significantly more often assigned to the stable medium compliance class (β = 0.33, *p* < .001), compared to the decreasing low compliance class and participants with higher education status were more often assigned to the stable high compliance class (β = 0.28, *p* = .004). However, these associations were not consistent across compliance classes. Neither gender, affective well-being, cognitive well-being, depressive symptoms nor anxiety symptoms were significantly associated with compliance class membership (see Table 4). Exploratory replication analyses, with the “decreasing high” compliance class as reference group, identified no consistent predictors for the compliance classes either (see supplementary Table S4).

**Table 4:** Association between baseline predictors and compliance class membership.

Affective well-being	Stable High compliance (vs. Low)			Stable Medium compliance (vs. Low)			Decreasing High compliance (vs. Low)		
	**β**	CI (2.5 %; 97.5 %)	*p*	**β**	CI (2.5 %; 97.5 %)	*p*	**β**	CI (2.5 %; 97.5 %)	*p*
Female gender	0.14	−0.06; 0.33	0.172	0.09	−0.09; 0.27	0.339	0.08	−0.08; 0.23	0.323
Age	0.08	−0.12; 0.28	0.449	0.32	0.12; 0.51	**0.001**	−0.11	−0.29; 0.07	0.239
Education	0.28	0.09; 0.48	**0.004**	0.20	0.02; 0.39	0.033	0.09	−0.06; 0.26	0.233
Affective well-being	−0.06	−0.26; 0.13	0.535	0.04	−0.14; 0.23	0.632	−0.12	−0.29; 0.04	0.148
COVID-stringency	0.29	0.06; 0.52	0.014	−0.09	−0.29; 0.10	0.356	−0.20	−0.37 −0.03	0.018


Cognitive well-being									

	**β**	CI (2.5 %; 97.5 %)	*p*	**β**	CI (2.5 %; 97.5 %)	*p*	**β**	CI (2.5 %; 97.5 %)	*p*

Female gender	0.14	−0.06; 0.33	0.165	0.09	−0.09; 0.27	0.319	0.08	−0.08; 0.23	0.326
Age	0.08	−0.12; 0.29	0.419	0.33	0.13; 0.52	**0.001**	−0.11	−0.29; 0.08	0.262
Education	0.28	0.09; 0.47	**0.004**	0.20	0.02; 0.39	0.034	0.09	−0.063; 0.26	0.235
Cognitive well-being	−0.03	−0.22; 0.17	0.793	0.08	−0.11; 0.27	0.425	−0.09	−0.25; 0.08	0.309
COVID-stringency	0.29	0.07; 0.52	0.012	−0.09	−0.28; 0.11	0.381	−0.19	−0.36; −0.03	0.022


Depressive symptoms									

	**β**	CI (2.5 %; 97.5 %)	*p*	**β**	CI (2.5 %; 97.5 %)	*p*	**β**	CI (2.5 %; 97.5 %)	*p*

Female gender	0.16	−0.04; 0.36	0.115	0.11	−0.08; 0.29	0.256	0.08	−0.08; 0.23	0.332
Age	0.11	−0.09; 0.31	0.294	0.33	0.14; 0.52	**0.001**	−0.09	−0.27; 0.09	0.319
Education	0.27	0.07; 0.46	**0.007**	0.18	−0.00; 0.37	0.053	0.10	−0.06; 0.26	0.214
Depression	−0.11	−0.31; 0.09	0.265	−0.15	−0.34; 0.05	0.140	0.06	−0.10; 0.23	0.449
COVID-stringency	0.32	0.09; 0.54	**0.007**	−0.08	−0.27; 0.11	0.419	−0.19	−0.35; −0.02	0.026


Anxiety symptoms									

	**β**	CI (2.5 %; 97.5 %)	*p*	**β**	CI (2.5 %; 97.5 %)	*p*	**β**	CI (2.5 %; 97.5 %)	*p*

Female gender	0.16	−0.04; 0.36	0.108	0.10	−0.08; 0.28	0.292	0.07	−0.08; 0.23	0.364
Age	0.12	−0.08; 0.32	0.247	0.33	0.14; 0.53	**0.001**	−0.10	−0.29; 0.08	0.272
Education	0.28	0.09; 0.47	**0.005**	0.20	0.02; 0.39	0.033	0.09	−0.07; 0.25	0.259
Anxiety	−0.12	−0.32; 0.08	0.228	−0.09	−0.27; 0.10	0.376	0.08	−0.09; 0.24	0.379
COVID-stringency	0.32	0.09; 0.55	**0.006**	−0.09	−0.28; 0.10	0.366	−0.19	−0.35; −0.03	0.024

*Note.* Bold font indicates significance after applying FDR correction.

### 3.4 Association between compliance classes and mental health trajectories

**Fig. 2:**
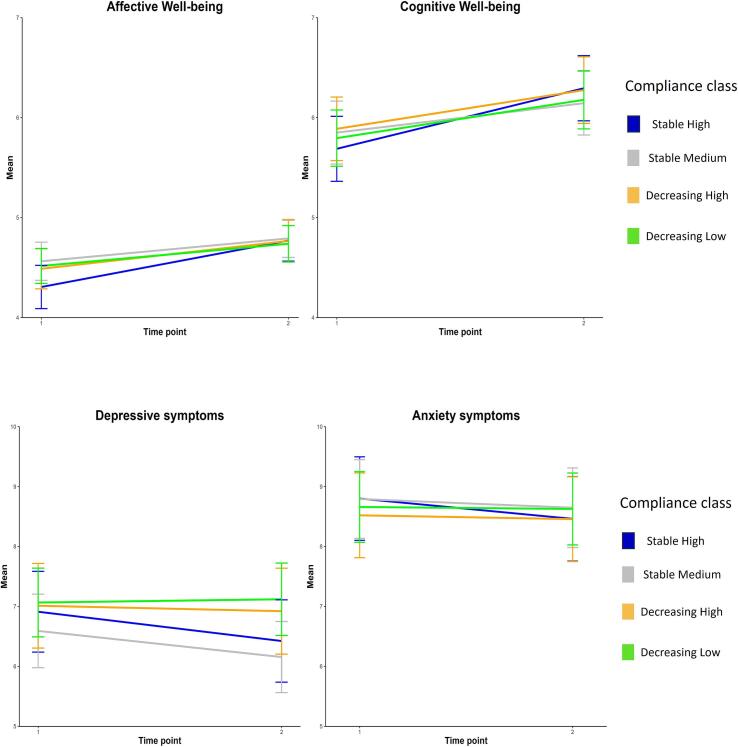
Changes in affective and cognitive well-being and symptoms of depression and anxiety, between compliance classes. *Note.* Lines represent mean levels per class; Whiskers represent 95 % confidence intervals.

Our third aim was to assess whether ESM compliance patterns were related to changes in affective and cognitive well-being, depressive symptoms, and anxiety symptoms due to the intervention. Multilevel models with two time points (baseline and follow-up) nested in participants revealed that participants significantly improved in affective (t(1435) = 6.72, p < .001) and cognitive well-being (t(1435) = 5.96, p < .001) between baseline and follow-up. Depressive symptoms (t(1425) = −2.63, *p* = .011) significantly decreased across the entire sample, while anxiety symptoms (t(1413) = −1.38, *p* = .193) did not significantly change. When comparing mean levels and confidence intervals between compliance classes, there were no differences in the development of affective and cognitive well-being, and depressive or anxiety symptoms between the classes (see Fig. 2). Pairwise comparisons between the groups also did not reveal any significant differences in changes in well-being, depressive, and anxiety symptoms between baseline and follow-up (see supplementary Table S5). An overview of the multilevel models can be found in supplementary Table S6.

## 4 Discussion

We aimed to further understand young people's ESM compliance in the Grow It! mHealth app (Dietvorst et al., 2022), its association with participant characteristics, and a potential intervention effect of self-monitoring via ESM on well-being and mental health. We identified four distinct patterns of ESM compliance in Grow It! users. However, aside from a weak association with age – older participants were more likely to show stable medium compliance – and education – participants following higher education were more likely to show stable high compliance – compliance patterns were unrelated to baseline participant characteristics. Contrary to our hypothesis, changes in well-being and mental health outcomes between baseline and follow-up, and therefore a potential intervention effect, were also unrelated to compliance patterns. Therefore, despite identifying distinct ESM compliance patterns among young people, we could not establish clear predictors and outcomes.

### 4.1 Identification of compliance patterns

As hypothesized (H1), we identified four distinct compliance patterns; one group of young people consistently completed most surveys (*mean overall compliance* = 78.8 %), one group steadily completed about half of the surveys (*mean overall compliance* = 50.1 %), one group initially completed most surveys with compliance strongly decreasing over time (*mean overall compliance* = 30.9 %) and one group initially completed few surveys which also decreased over time (*mean overall compliance* = 13.2 %).

Previous studies identified an overall trend of young people's ESM compliance decreasing over time (e.g. Connelly et al., 2012; Stinson et al., 2008). However, groups of young people with different compliance patterns have not been identified before. This has important implications. Despite all participants following the same ESM design, clusters of participants already started at different compliance levels with these differences expanding over time. These pronounced compliance differences between participants can therefore not be explained by study burden alone. Instead, we need to examine what other external, internal, and contextual factors influence compliance patterns.

First, 43 % of young people (*N* = 498) showed decreasing low compliance, indicating that many participants lacked motivation to complete ESM surveys in the first place. This could be because of little external motivators (e.g., no direct monetary incentive; Heron et al., 2017), but also because they simply forgot to respond. While many ESM studies actively remind participants to respond (van Dalen et al., 2024), we did not contact participants during the study period, aside from automated online reminders 40 min after each prompt. This could have influenced compliance but can – along with the lack of an external reward - provide important insights for the clinical implementation of ESM, as mHealth interventions aim to be effective in real-life settings and with little to no monitoring.

Secondly, identifying participants who started with high compliance but decreased strongly over time (*N* = 272, 24 %) indicates that Grow It! might not have encouraged long-term engagement. In ESM studies, participants sometimes abruptly stop complying when reaching the threshold for an external reward (Wrzus and Neubauer, 2023). However, we identified a steady decline in compliance instead. Bülow et al. (2025) suggest that intrinsic motivators like gaining self-insight or helping science could keep young people engaged with ESM protocols. Some studies, for example, inform participants beforehand about important societal implications of their research (van Roekel et al., 2019). Including a personal mood profile as an intrinsic motivator did not improve ESM compliance in Grow It! (Dietvorst et al., 2024b). Future ESM intervention studies should therefore explore additional intrinsic motivators to prevent decreasing compliance in similar mHealth apps.

Finally, contextual factors could affect ESM compliance patterns. The disruption of daily routines during the COVID-19 pandemic (Stavridou et al., 2020) could have impaired young people's ability and motivation to comply with the ESM intervention in our sample. Therefore, replicating these different compliance classes without COVID restrictions is important.

### 4.2 Participant characteristics and compliance

Although we identified different ESM compliance classes, these groups of young people did not differ in gender, baseline affective and cognitive well-being, or depressive and anxiety symptoms. Meanwhile, older participants were more likely to show stable medium compliance compared to decreasing low compliance, while participants in higher education were more likely to show stable high compliance. However, these small effects were not consistent across classes, with other compliance classes not showing significant differences to the decreasing low compliance class. Education is often not assessed as a predictor for ESM compliance (Rintala et al., 2019), so future studies should further examine this small finding. Overall, our findings indicate that participant characteristics are mostly unrelated to young people's ESM compliance - similar to the meta-analysis of Wen et al. (2017). This suggests that young people with higher depression and anxiety levels at baseline could comply with a mHealth intervention at similar levels as other participants. However, other characteristics that could affect ESM compliance might not have been evaluated. Murray et al. (2023) found higher antisocial behavior and lower self-control associated with lower ESM compliance in young adults, while depressive and anxiety symptoms were not. With self-control increasing during adolescence (Zondervan-Zwijnenburg et al., 2020), this would correspond to older participants showing slightly more stable compliance in our study. Understanding which additional participant characteristics affect ESM compliance among young people could help personalize ESM designs.

### 4.3 ESM compliance and the intervention's efficacy

The Grow It! app has been shown to positively affect well-being (Dietvorst et al., 2023). However, contrary to our hypothesis (H2) that young people in the highest compliance class showed the strongest increase in well-being, all compliance classes showed similar changes in affective and cognitive well-being from baseline to follow-up. Compliance patterns were also unrelated to changes in depressive or anxiety symptoms. This indicates that the positive intervention effect of Grow It! was independent of ESM compliance. Therefore, Grow It! might potentially work differently for different participants, as the effect on well-being and mental health could depend on different aspects of the intervention. Some young people might have profited from self-monitoring via ESM, while others profited more from the challenges, i.e., the other core aspect of Grow It!.

This potential positive effect of combined intervention elements in Grow It! on well-being signals the need for personalizing ESM-based mHealth interventions. Some researchers recommend implementing mHealth intervention components at moments where participants might benefit the most (Bidargaddi et al., 2020). These “Just-in-time adaptive interventions” (Nahum-Shani et al., 2018) could be particularly useful in mHealth interventions with an ESM component. In Grow It!, one could measure time point specific distress via ESM and implement suitable challenges accordingly to improve the intervention's effect on well-being and mental health outcomes. Other studies suggest personalizing ESM items that participants receive, based on the participant's mood profiles and feedback (Riese et al., 2021). Participants can then select ESM items depending on which aspects they perceive as the most relevant. Approaches like these could enable more targeted intervention effects on well-being and mental health, reduce design burden, and increase long-term engagement with the intervention.

### 4.4 Limitations

The average compliance across participants was low (33.8 %), compared to usual levels of adolescent ESM compliance (78 %; Wen et al., 2017, 74 %; van Roekel et al., 2019). With a disproportionately large “decreasing low” compliance class, potential effects of baseline predictors became harder to compare between classes. Future studies that examine ESM compliance patterns should aim for higher overall compliance levels.

Furthermore, many participants (*N* = 377; 33 %) did not complete follow-up surveys. While young people who dropped out did not significantly differ in demographic characteristics and outcome variables from other participants (Dietvorst et al., 2023), the dropout makes it harder to attribute changes in well-being and mental health to their compliance. Young people whose well-being and mental health improved might have felt no need to continue participating and therefore showed lower compliance. In contrast, young people who experienced no improvements yet could have been more motivated to continue. The ESM protocol explicitly being a part of an intervention could also have affected participants´ expectations and compliance levels. Some participants might have dropped out because they did not perceive improvements in their well-being or additional insights into their mood, while they would have continued ESM protocols without these expectations. Similarly, ESM compliance might have increased in participants who perceived improvements caused by other Grow It! components, like the challenges. Initial user evaluations revealed that most Grow It! users would recommend the app and that user rating was not related to ESM compliance or changes in well-being (Dietvorst et al., 2023). Also, de Vries et al. (in preparation) found only slightly higher compliance to the Grow It! challenges (*Mean* = 43 %; range = 5–100 %) than to the ESM protocol. Still, this makes it harder to generalize our findings to ESM studies without an intervention aspect. To better understand these dynamics, future studies should explicitly ask participants about reasons for non-compliance to ESM and to other intervention components.

Despite examining a large population, our sample was rather homogenous (79 % female; 92 % Dutch), due to our open recruitment procedure. This impairs the generalizability of our findings to other populations (Haeffel and Cobb, 2022). Moreover, participants showed low depressive and anxiety symptoms and high levels of well-being. This might have disguised potentially existing effects of these participant characteristics on ESM compliance and enabled floor and ceiling effects when assessing changes in well-being and mental health (Morton and Torgerson, 2005). Future ESM studies that assess intervention effects should ensure heterogeneity in their sample, especially in participant characteristics of interest.

Finally, we used a pre-post design without a control condition. This complicates the attribution of improvements in well-being and mental health outcomes to the intervention itself. However, the positive effects have been replicated in three independent cohorts during the COVID-19 pandemic (Mens et al., 2022; Dietvorst et al., 2024b), while during the pandemic an overall decline in young people's mental health was observed (Racine et al., 2021). Still, other explanations for the improvements in well-being and mental health, like regression to the mean are possible (Morton and Torgerson, 2005). To disentangle the intervention effects that different aspects of Grow It! might have, and to examine contributing external factors, a randomized control trial study currently examines the intervention effect of Grow It! in more detail (Grow It, n.d.; ISRCTN - ISRCTN17883961).

### 4.5 Conclusion

The present study identified four patterns of ESM compliance in young people. However, we could not establish clear predictors of compliance patterns or an association between compliance and an ESM intervention's effect on mental health and well-being. Our identification of young people showing high, medium, or decreasing compliance across multiple weeks of ESM can guide future approaches in personalizing ESM schedules or implementing interventions such as CBT-based challenges at the most beneficial moments (i.e., Just-in-time adaptive interventions). Despite not identifying age, gender, education, well-being, or mental health as clear predictors for compliance, future studies could use our insights and examine other participant characteristics like self-control. Furthermore, researchers should examine motivators like personalized feedback or monetary rewards that might affect compliance patterns. Finally, with this study being unique in examining the association between ESM compliance patterns and a potential intervention effect, future research can investigate whether personalizing ESM designs could strengthen the intervention effects of mHealth apps for improving well-being and mental health outcomes in young people.

## Funding

This project is part of the research project “Stress in Action”. It is financially supported by the 10.13039/501100012415Dutch Research Council and the Dutch Ministry of Education, Culture and Science (NWO gravitation grant number 024.005.010).

It is also part of the PROTECt ME project, funded by the Convergence, the alliance between Erasmus Medical Centre Rotterdam, Erasmus University Rotterdam and Delft University of Technology.

This project uses secondary data. The original study is funded by 10.13039/501100001826Netherlands Organisation for Health Research and Development ‘Juiste Zorg Op De Juiste Plek’ (ZonMw JZOJP) together with the 10.13039/501100003246Netherlands Organisation for Scientific Research (NWO; project number: 440.20.006), the 10.13039/501100009480Stichting Vrienden van het Sophia (SSWO; project number: B18–05), Gemeente Rotterdam (project numbers: SUB.20.03.00149.SBSA and 40258362) and the Dutch National Research Agenda (NWA/eHealth Junior consortium; project number: 1292.19.226).

## Declaration of competing interest

The authors declare that they have no known competing financial interests or personal relationships that could have appeared to influence the work reported in this paper.

## Appendix A Supplementary data

Supplementary material Image 1
