# Supplementary material for: Young people's compliance with the Experience Sampling Method (ESM): Examining patterns, predictors and associations with well-being and mental health
Source: Internet Interv. 2025 Jul 8;41:100859. doi: 10.1016/j.invent.2025.100859 (PMC12275891; doi:10.1016/j.invent.2025.100859)
Supplement: Supplementary file 1 — Supplementary material [file mmc1.docx]

**Supplementary material**

**Supplementary 1: Overview and additional analyses in the three weeks cohort**

**Table S1.** Correlation between all measured baseline predictors and compliance in the three weeks cohort

| Variable | 1 | 2 | 3 | 4 | 5 | 6 | 7 | 8 | 9 | 10 |
| --- | --- | --- | --- | --- | --- | --- | --- | --- | --- | --- |
| 1. Compliance (%) | - |  |  |  |  |  |  |  |  |  |
| 2. Age | 0.07 | - |  |  |  |  |  |  |  |  |
| 3. Education | 0.08 | -0.08 | - |  |  |  |  |  |  |  |
| 4. Aff. well-being | -0.04 | -0.31 | 0.10 | - |  |  |  |  |  |  |
| 5. Cog. well-being | -0.02 | -0.35 | 0.11 | **0.82** | - |  |  |  |  |  |
| 6. Depression | -0.04 | 0.24 | -0.21 | **-0.68** | **-0.69** | - |  |  |  |  |
| 7. Anxiety | -0.02 | 0.33 | -0.11 | **-0.57** | **-0.58** | **0.72** | - |  |  |  |
| 8. Loneliness | 0.00 | 0.30* | -0.09 | **-0.54** | **-0.50** | **0.59** | **0.54** | - |  |  |
| 9. Adapt. Coping | -0.01 | -0.02 | 0.04 | 0.29 | 0.28 | -0.34 | -0.21 | -0.26 | - |  |
| 10. Mal. Coping | -0.02 | 0.09 | -0.08 | -0.29 | -0.31 | 0.37 | 0.36 | 0.24 | -0.14 | - |

*Note.* Bold font indicates significance after implementing FDR correction; Aff. well-being = Affective well-being; Cog. well-being = Cognitive well-being; Adapt. Coping; = Adaptive coping; Maladapt. coping = Maladaptive coping

**Table S2.** Multinomial regression model including all measured baseline predictors

|  |  | Stable High compliance (vs. Low) |  |  | Stable Medium compliance (vs. Low) |  |  | Decreasing High compliance (vs. Low) |  |
| --- | --- | --- | --- | --- | --- | --- | --- | --- | --- |
|  | **β** | CI (2.5%; 97.5%) | *p* | **β** | CI (2.5%; 97.5%) | *p* | **β** | CI (2.5%; 97.5%) | *p* |
| Female gender | 0.19 | -0.01; 0.39 | .064 | 0.11 | -0.08; 0.29 | .268 | 0.12 | -0.05; 0.28 | .170 |
| Age | 0.07 | -0.14; 0.29 | .520 | 0.29 | 0.07; 0.50 | .009 | -0.17 | -0.37; 0.04 | .107 |
| Education | 0.27 | 0.07; 0.47 | .009 | 0.19 | -0.01; 0.39 | .059 | 0.11 | -0.06; 0.28 | .218 |
| Aff. well-being | -0.31 | -0.66; 0.04 | .080 | -0.11 | -0.45; 0.23 | .538 | -0.22 | -0.52; 0.08 | .147 |
| Cog. well-being | -0.04 | -0.39; 0.31 | .818 | 0.08 | -0.27; 0.42 | .665 | 0.05 | -0.26; 0.35 | .766 |
| Depression | -0.21 | -0.56; 0.13 | .224 | -0.46 | -0.81; -0.11 | .010 | -0.19 | -0.49; 0.11 | .204 |
| Anxiety | -0.13 | -0.42; 0.16 | .388 | -0.14 | -0.42; 0.14 | .334 | -0.08 | -0.33; 0.17 | .518 |
| Loneliness | -0.09 | -0.35; 0.16 | .447 | 0.36 | 0.11; 0.61 | .005 | 0.12 | -0.10; 0.35 | .283 |
| Treatment | 0.04 | -0.18; 0.26 | .712 | 0.16 | -0.05; 0.38 | .142 | 0.20 | 0.02; 0.39 | .028 |
| Waitlist | -0.03 | -0.28; 0.22 | .832 | 0.29 | 0.11; 0.47 | **.001** | 0.20 | 0.03; 0.37 | .023 |
| Adapt. coping | 0.01 | -0.19; 0.21 | .902 | -0.02 | -0.22; 0.18 | .843 | 0.02 | -0.15; 0.19 | .804 |
| Maladapt. coping | -0.05 | -0.25; 0.15 | .630 | 0.10 | -0.09; 0.30 | .319 | 0.06 | -0.11; 0.24 | .472 |
| COVID stringency | 0.27 | 0.03; 0.51 | .027 | -0.159 | -0.37; 0.06 | .145 | -0.254 | -0.43; -0.08 | .005 |

*Note.* Bold font indicates significance after implementing FDR correction; Aff. well-being = Affective well-being; Cog. well-being = Cognitive well-being; Adapt. coping; = Adaptive coping

**Table S3.** Overview of latent class growth analyses and model selection for the three weeks cohort

|  | loglik | npm | AIC | BIC | entropy | posterior probability | %class1 | %class2 | %class3 | %class4 | %class5 |
| --- | --- | --- | --- | --- | --- | --- | --- | --- | --- | --- | --- |
| 1-class model | -82027.9 | 3 | 164061 | 164077 | 1.00 | - | 100 |  |  |  |  |
| 2-class model | -67472.7 | 6 | 134957 | 134987 | 0.99 | > 99% | 70.94 | 29.05 |  |  |  |
| 3-class model | -60402.6 | 9 | 120823 | 120868 | 0.96 | > 96% | 21.51 | 31.08 | 47.41 |  |  |
| **4-class model** | **-59070.5** | **12** | **118225.6** | **118165.6** | **0.96** | **> 96%** | **15.45** | **16.85** | **23.79** | **43.89** |  |
| 5-class model | -58292.2 | 15 | 116614.5 | 116690.1 | 0.96 | > 96% | 24.14 | 7.63 | 40.38 | 15.18 | 12.64 |

*Note.* loglik = Log-Likelihood of model; npm = number of parameters; entropy = degree of class separation from 0 to 1; posterior probability = minimum likelihood of participants to end up in their assigned class; %class1 to %class5 = percentage of participants assigned to that particular class; Bold font indicates the selected model.

**Table S4.** Exploratory multinomial regressions with the “decreasing high” class as reference group

| Affective well-being |  | | Stable High compliance (vs. Decreasing High) |  |  | Stable Medium compliance (vs. Decreasing High) |  |  | Decreasing Low compliance (vs. Decreasing High) |  |
| --- | --- | --- | --- | --- | --- | --- | --- | --- | --- | --- |
|  | | **β** | CI (2.5%; 97.5%) | *p* | **β** | CI (2.5%; 97.5%) | *p* | **β** | CI (2.5%; 97.5%) | *p* |
| Female gender | | 0.14 | -0.38; 0.67 | .016 | 0.03 | -0.47; 0.52 | .921 | -0.19 | -0.57; 0.19 | .323 |
| Age | | 0.19 | -0.04; 0.42 | .592 | 0.43 | 0.21; 0.65 | **<.001** | 0.11 | -0.07; 0.29 | .239 |
| Education | | 0.19 | -0.03; 0.39 | .105 | 0.10 | -0.10; 0.31 | .327 | -0.09 | -0.26; 0.06 | .233 |
| Affective Well-being | | 0.06 | -0.15; 0.28 | .090 | 0.17 | -0.04; 0.38 | .115 | 0.12 | -0.04; 0.29 | .148 |
| COVID-stringency | | 0.49 | 0.25; 0.73 | **<.001** | 0.11 | -0.10; 0.32 | .310 | 0.20 | 0.04; 0.37 | .018 |

Cognitive well-being

|  | **β** | CI (2.5%; 97.5%) | *p* | **β** | CI (2.5%; 97.5%) | *p* | **β** | CI (2.5%; 97.5%) | *p* |
| --- | --- | --- | --- | --- | --- | --- | --- | --- | --- |
| Female gender | 0.15 | -0.38; 0.68 | .575 | 0.04 | -0.46; 0.53 | .887 | -0.19 | -0.57; 0.19 | .326 |
| Age | 0.19 | -0.04; 0.42 | .105 | 0.43 | 0.21; 0.65 | **<.001** | 0.11 | -0.08; 0.29 | .262 |
| Education | 0.18 | -0.03; 0.39 | .091 | 0.10 | -0.10; 0.31 | .330 | -0.09 | -0.26; 0.06 | .235 |
| Cognitive Well-being | 0.06 | -0.16; 0.28 | .585 | 0.17 | -0.05; 0.38 | .127 | 0.09 | -0.08; 0.25 | .309 |
| COVID-stringency | 0.49 | 0.25; 0.73 | **<.001** | 0.10 | -0.10; 0.31 | .325 | 0.19 | 0.03; 0.36 | .022 |

Depressive symptoms

|  | **β** | CI (2.5%; 97.5%) | *p* | **β** | CI (2.5%; 97.5%) | *p* | **β** | CI (2.5%; 97.5%) | *p* |
| --- | --- | --- | --- | --- | --- | --- | --- | --- | --- |
| Female gender | 0.20 | -0.33; 0.73 | .459 | 0.07 | -0.43; 0.57 | .782 | -0.19 | -0.57; 0.19 | .332 |
| Age | 0.19 | -0.03; 0.42 | .082 | 0.42 | 0.20; 0.64 | **<.001** | 0.09 | -0.09; 0.27 | .319 |
| Education | 0.16 | -0.05; 0.38 | .138 | 0.08 | -0.13; 0.29 | .445 | -0.10 | -0.26; 0.06 | .214 |
| Depression | -0.18 | -0.39; 0.04 | .112 | -0.21 | -0.42; 0.003 | .054 | -0.06 | -0.23; 0.10 | .449 |
| COVID-stringency | 0.50 | 0.26; 0.74 | **<.001** | 0.11 | -0.10; 0.31 | .316 | 0.19 | 0.02; 0.35 | .026 |

Anxiety symptoms

|  | **β** | CI (2.5%; 97.5%) | *p* | **β** | CI (2.5%; 97.5%) | *p* | **β** | CI (2.5%; 97.5%) | *p* |
| --- | --- | --- | --- | --- | --- | --- | --- | --- | --- |
| Female gender | 0.22 | -0.31; 0.75 | .417 | 0.06 | -0.43; 0.56 | .802 | -0.18 | -0.56; 0.21 | .364 |
| Age | 0.22 | -0.01; 0.45 | .056 | 0.43 | 0.21; 0.66 | **<.001** | 0.10 | -0.08; 0.29 | .272 |
| Education | 0.19 | -0.03; 0.40 | .089 | 0.11 | -0.09; 0.32 | .304 | -0.09 | -0.25; 0.07 | .259 |
| Anxiety | -0.19 | -0.41; 0.02 | .077 | -0.16 | -0.37; 0.05 | .133 | -0.08 | -0.24; 0.09 | .379 |
| COVID-stringency | 0.51 | 0.27; 0.75 | **<.001** | 0.09 | -0.11; 0.31 | .349 | 0.19 | 0.03; 0.35 | .024 |

*Note.* Bold font indicates significance after applying FDR correction

**Table S5.** Pairwise comparisons between compliance classes in developments of well-being, depression, and anxiety

| Affective well-being | |  |  |  |  |  | Cognitive well-being | |  |  |  |  |
| --- | --- | --- | --- | --- | --- | --- | --- | --- | --- | --- | --- | --- |
| Time point | Class 1 | Class 2 | n1 | n2 | *p* |  | Time point | Class 1 | Class 2 | n1 | n2 | *p* |
| 1 | St. High | St. Med. | 170 | 174 | .082 |  | 1 | St. High | St. Med. | 170 | 174 | .489 |
| 1 | St. High | Decr.High | 170 | 170 | .220 |  | 1 | St. High | Decr.High | 170 | 170 | .397 |
| 1 | St.Med. | Decr. High | 174 | 170 | .612 |  | 1 | St.Med. | Decr. High | 174 | 170 | .872 |
| 1 | St. High | Decr. Low | 170 | 244 | .124 |  | 1 | St. High | Decr. Low | 170 | 244 | .623 |
| 1 | St. Med. | Decr. Low | 174 | 244 | .731 |  | 1 | St. Med. | Decr. Low | 174 | 244 | .797 |
| 1 | Decr. High | Decr. Low | 170 | 244 | .837 |  | 1 | Decr. High | Decr. Low | 170 | 244 | .668 |
| 2 | St. High | St. Med. | 170 | 174 | .887 |  | 2 | St. High | St. Med. | 170 | 174 | .534 |
| 2 | St. High | Decr. High | 170 | 170 | .978 |  | 2 | St. High | Decr. High | 170 | 170 | .939 |
| 2 | St. Med. | Decr. High | 174 | 170 | .866 |  | 2 | St. Med. | Decr. High | 174 | 170 | .588 |
| 2 | St. High | Decr. Low | 170 | 244 | .818 |  | 2 | St. High | Decr. Low | 170 | 244 | .604 |
| 2 | St. Med. | Decr. Low | 174 | 244 | .700 |  | 2 | St. Med. | Decr. Low | 174 | 244 | .879 |
| 2 | Decr. High | Decr. Low | 170 | 244 | .842 |  | 2 | Decr. High | Decr. Low | 170 | 244 | .665 |
| Depression |  |  |  |  |  |  | Anxiety |  |  |  |  |  |
| Time point | Class 1 | Class 2 | n1 | n2 | *p* |  | Time point | Class 1 | Class 2 | n1 | n2 | *p* |
| 1 | St. High | St. Med. | 170 | 174 | .509 |  | 1 | St. High | St. Med. | 170 | 174 | .989 |
| 1 | St. High | Decr.High | 170 | 170 | .837 |  | 1 | St. High | Decr.High | 170 | 170 | .579 |
| 1 | St.Med. | Decr. High | 174 | 170 | .387 |  | 1 | St.Med. | Decr. High | 174 | 170 | .586 |
| 1 | St. High | Decr. Low | 170 | 244 | .732 |  | 1 | St. High | Decr. Low | 170 | 244 | .762 |
| 1 | St. Med. | Decr. Low | 174 | 244 | .288 |  | 1 | St. Med. | Decr. Low | 174 | 244 | .772 |
| 1 | Decr. High | Decr. Low | 170 | 244 | .905 |  | 1 | Decr. High | Decr. Low | 170 | 244 | .764 |
| 2 | St. High | St. Med. | 170 | 174 | .589 |  | 2 | St. High | St. Med. | 170 | 174 | .714 |
| 2 | St. High | Decr. High | 170 | 170 | .320 |  | 2 | St. High | Decr. High | 170 | 170 | .993 |
| 2 | St. Med. | Decr. High | 174 | 170 | .125 |  | 2 | St. Med. | Decr. High | 174 | 170 | .710 |
| 2 | St. High | Decr. Low | 170 | 244 | .132 |  | 2 | St. High | Decr. Low | 170 | 244 | .729 |
| 2 | St. Med. | Decr. Low | 174 | 244 | .036 |  | 2 | St. Med. | Decr. Low | 174 | 244 | .965 |
| 2 | Decr. High | Decr. Low | 170 | 244 | .669 |  | 2 | Decr. High | Decr. Low | 170 | 244 | .725 |

*Note.* St. High = Stable High; St. Med. = Stable Medium; Decr. High = Decreasing High; Decr. Low = Decreasing Low

**Table S6.** Fixed effects in multilevel models of changes in well-being, depressive symptoms and anxiety between baseline (T1) and follow-up (T2).

| Aff. well-being (T2) |  |  | | |  | |  | |  | | |  | | |  |  |  |  |  |  |  |  |  |  |  |  |  |  |  |  |  |  |  |  |  |  |  |  |  |  |
| --- | --- | --- | --- | --- | --- | --- | --- | --- | --- | --- | --- | --- | --- | --- | --- | --- | --- | --- | --- | --- | --- | --- | --- | --- | --- | --- | --- | --- | --- | --- | --- | --- | --- | --- | --- | --- | --- | --- | --- | --- |
|  |  | Entire cohort | | | | | | | |  | | | Stable High | | | | |  | | Stable Medium | | | | |  | | | Decreasing High | | | |  | | | Decreasing Low | | | |  |  |
|  | B | | | *SE* | | *t* | | *p* | | | B | | | *SE* | | *t* | *p* | | B | | | *SE* | *t* | *p* | | B | | | *SE* | *t* | *p* | | B | | | *SE* | *t* | *p* | | |
| (Intercept) | | | 10.29 | 0.83 | | 12.33 | | <.001 | | | 10.44 | | | 2.17 | | 4.81 | <.001 | | | | 10.83 | 1.63 | 6.65 | <.001 | | | 11.29 | | 1.69 | 6.65 | <.001 | | | 9.79 | | 1.51 | 6.47 | <.001 | | |
| Aff. well-being (T1) | | | 0.31 | 0.05 | | 6.72 | | **<.001** | | | 0.47 | | | 0.09 | | 4.84 | **<.001** | | | | 0.27 | 0.09 | 2.74 | **.007** | | | 0.29 | | 0.09 | 3.34 | **.001** | | | 0.24 | | 0.09 | 2.81 | **.005** | | |
| Age | | | -0.08 | 0.01 | | -5.64 | | **<.001** | | | -0.08 | | | 0.02 | | -2.86 | .004 | | | | -0.04 | 0.02 | -1.54 | .113 | | | -0.06 | | 0.03 | -1.91 | .056 | | | -0.11 | | 0.02 | -4.63 | **<.001** | | |
| Female | | | -0.25 | 0.11 | | -2.42 | | **.017** | | | -0.46 | | | 0.24 | | -1.89 | .067 | | | | -0.32 | 0.22 | -1.42 | .161 | | | -0.11 | | 0.23 | -0.49 | .597 | | | -0.24 | | 0.18 | 0.17 | .172 | | |
| Education | | | 0.02 | 0.05 | | 0.41 | | .666 | | | 0.12 | | | 0.13 | | 0.93 | .348 | | | | -0.09 | 0.11 | -0.83 | .428 | | | -0.09 | | 0.14 | -0.61 | .533 | | | 0.09 | | 0.09 | 0.98 | .330 | | |
| Covid stringency | | | -0.05 | 0.01 | | -5.12 | | **<.001** | | | -0.05 | | | 0.02 | | -2.10 | .036 | | | | -0.06 | 0.02 | -3.02 | **.003** | | | -0.07 | | 0.02 | -3.23 | **.002** | | | -0.04 | | 0.02 | -2.20 | .028 | | |
| Cog. well-being (T2) | | |  |  | |  | |  | | |  | | |  | |  |  | | | |  |  |  |  | | |  | |  |  |  | | |  | |  |  |  | | |
|  | | | B | *SE* | | *t* | | *p* | | | B | | | *SE* | | *t* | *p* | | | | B | *SE* | *t* | *p* | | | B | | *SE* | *t* | *p* | | | B | | *SE* | *t* | *p* | | |
| (Intercept) | | | 15.40 | 1.33 | | 11.55 | | <.001 | | | 17.37 | | | 3.42 | | 5.09 | <.001 | | | | 15.93 | 2.71 | 5.87 | <.001 | | | 15.59 | | 2.65 | 5.86 | <.001 | | | 14.72 | | 2.39 | 6.15 | <.001 | | |
| Cog. well-being (T1) | | | 0.42 | 0.07 | | 5.96 | | **<.001** | | | 0.64 | | | 0.14 | | 4.49 | **<.001** | | | | 0.25 | 0.16 | 1.59 | 0.11 | | | 0.43 | | 0.14 | 3.12 | **.003** | | | 0.40 | | 0.13 | 3.00 | **.003** | | |
| Age | | | -0.15 | 0.02 | | -6.82 | | **<.001** | | | -0.13 | | | 0.05 | | -2.83 | **.005** | | | | -0.07 | 0.04 | 0.09 | 0.09 | | | -0.14 | | 0.05 | -2.68 | **.008** | | | -0.23 | | 0.03 | -5.99 | **<.001** | | |
| Female | | | -0.59 | 0.17 | | -3.46 | | **<.001** | | | -0.84 | | | 0.39 | | -2.15 | .035 | | | | -0.94 | 0.37 | -2.50 | **0.01** | | | -0.37 | | 0.36 | -1.02 | .318 | | | -0.49 | | 0.28 | -1.76 | .080 | | |
| Education | | | 0.04 | 0.09 | | 0.39 | | .693 | | | 0.07 | | | 0.21 | | 0.34 | .734 | | | | -0.23 | 0.19 | -1.24 | 0.22 | | | 0.03 | | 0.22 | 0.14 | .888 | | | 0.16 | | 0.15 | 1.06 | .291 | | |
| Covid stringency | | | -0.08 | 0.01 | | -4.89 | | **<.001** | | | -0.11 | | | 0.04 | | -2.62 | **.009** | | | | -0.09 | 0.03 | -2.63 | **.009** | | | -0.09 | | 0.03 | -2.71 | **.007** | | | -0.31 | | 0.15 | -2.07 | .041 | | |
| Depression (T2) | | |  |  | |  | |  | | |  | | |  | |  |  | | | |  |  |  |  | | |  | |  |  |  | | |  | |  |  |  | | |
|  | | | B | *SE* | | *t* | | *p* | | | B | | | *SE* | | *t* | *p* | | | | B | *SE* | *t* | *p* | | | B | | *SE* | *t* | *p* | | | B | | *SE* | *t* | *p* | | |
| (Intercept) | | | -6.53 | 2.96 | | -2.20 | | .027 | | | -11.7 | | | 7.54 | | -1.48 | .139 | | | | -8.90 | 5.64 | -1.58 | .116 | | | -7.94 | | 6.21 | -1.28 | .202 | | | -4.85 | | 5.37 | -0.90 | .367 | | |
| Depression (T1) | | | -0.27 | 0.10 | | -2.63 | | **.011** | | | -0.51 | | | 0.19 | | -2.68 | **.008** | | | | -0.42 | 0.20 | -2.07 | .044 | | | -0.26 | | 0.21 | -1.22 | .273 | | | 0.01 | | 0.21 | 0.03 | .979 | | |
| Age | | | 0.11 | 0.05 | | 2.28 | | **.022** | | | 0.09 | | | 0.10 | | 0.89 | .361 | | | | -0.04 | 0.09 | -0.39 | .695 | | | 0.23 | | 0.12 | 1.91 | .058 | | | 0.21 | | 0.08 | 2.48 | **.014** | | |
| Female | | | 1.48 | 0.38 | | 3.87 | | **<.001** | | | 1.83 | | | 0.86 | | 2.13 | .037 | | | | 2.03 | 0.78 | 2.61 | **.010** | | | 1.08 | | 0.86 | 1.26 | .214 | | | 1.44 | | 0.64 | 2.26 | .025 | | |
| Education | | | -0.77 | 0.21 | | -3.71 | | **<.001** | | | -1.09 | | | 0.46 | | -2.34 | **.020** | | | | -0.09 | 0.39 | -0.23 | .817 | | | -0.45 | | 0.52 | -0.86 | .391 | | | -1.09 | | 0.34 | -3.16 | **.002** | | |
| Covid stringency | | | 0.15 | 0.03 | | 4.04 | | **<.001** | | | 0.22 | | | 0.09 | | 2.36 | **.019** | | | | 0.19 | 0.07 | 2.54 | **.012** | | | 0.14 | | 0.08 | 1.83 | .069 | | | 0.12 | | 0.07 | 1.77 | .078 | | |
| Anxiety (T2) | | |  |  | |  | |  | | |  | | |  | |  |  | | | |  |  |  |  | | |  | |  |  |  | | |  | |  |  |  | | |
|  | | | B | *SE* | | *t* | | *P* | | | B | | | *SE* | | *t* | *p* | | | | B | *SE* | *t* | *p* | | | B | | *SE* | *t* | *p* | | | B | | *SE* | *t* | *p* | | |
| (Intercept) | | | -13.4 | 2.95 | | -4.56 | | <.001 | | | -13.3 | | | 8.07 | | -1.65 | .098 | | | | -16.3 | 5.88 | -2.78 | .006 | | | -13.3 | | 5.97 | -2.24 | .026 | | | -12.5 | | 5.19 | -2.43 | .016 | | |
| Anxiety (T1) | | | -0.18 | 0.13 | | -1.38 | | .193 | | | -0.33 | | | 0.26 | | -1.29 | .189 | | | | -0.21 | 0.24 | -0.86 | .417 | | | -0.21 | | 0.26 | -0.80 | .508 | | | -0.01 | | 0.25 | -0.05 | .964 | | |
| Age | | | 0.23 | 0.04 | | 4.73 | | **<.001** | | | 0.17 | | | 0.11 | | 1.52 | .124 | | | | 0.21 | 0.09 | 2.23 | .027 | | | 0.25 | | 0.11 | 2.16 | **.032** | | | 0.28 | | 0.08 | 3.45 | **<.001** | | |
| Female | | | 1.84 | 0.38 | | -4.85 | | **<.001** | | | 1.94 | | | 0.92 | | 2.11 | .040 | | | | 2.11 | 0.81 | 2.59 | **.011** | | | 1.32 | | 0.83 | 1.59 | .110 | | | 2.06 | | 0.61 | 3.35 | **<.001** | | |
| Education | | | -0.17 | 0.20 | | -0.80 | | .415 | | | -0.03 | | | 0.50 | | -0.08 | .933 | | | | 0.09 | 0.40 | 0.23 | .829 | | | -0.19 | | 0.51 | -0.37 | .716 | | | -0.35 | | 0.33 | -1.04 | .300 | | |
| Covid stringency | | | 0.22 | 0.04 | | 5.72 | | **<.001** | | | 0.22 | | | 0.10 | | 2.21 | .028 | | | | 0.25 | 0.08 | 2.93 | **.002** | | | 0.22 | | 0.08 | 3.27 | **.004** | | | 0.20 | | 0.07 | 2.95 | **.004** | | |

Note. Bold font indicates significance after applying FDR correction; Aff. well-being = Affective well-being; Cog. well-being = Cognitive well-being; We used the values of cognitive and affective well-being, depression, and anxiety at T1 to predict the respective values at T2. Coefficients indicate the significance of change in the variable from T1 to T2 when controlled for the other variables.

**Supplementary 2: Results of the six weeks cohort**

Another cohort of adolescents used the Grow It! app for six weeks instead of three. In the following supplements, we report about the results of this cohort when implementing the same analysis plan as for the three weeks cohorts.

**Table S7.** Sample characteristics and descriptive statistics of the six weeks cohort

|  | Baseline  (*N* = 496)  M (SD) or % | Follow-up  (*N* = 345)  M (SD) or % |
| --- | --- | --- |
| **Demographic variables** |  |  |
| Age | 16.32 (2.99) | 16.36 (2.96) |
| Gender (% female) | 76% | 76% |
| Ethnicity | 89% Dutch | 88% Dutch |
|  | 9% Mixed | 9% Mixed |
|  | 2% Other | 3% Other |
| Education level | 4% Primary | 4% Primary |
|  | 13%Low | 11% Low |
|  | 22% Middle | 23% Middle |
|  | 61% High | 62% High |
| **Study outcomes** |  |  |
| ESM Compliance % | 24.2% (22.8) | 29.8% (25.55) |
| Affective Well-being | 4.90 (1.29) | 5.31 (1.28) |
| Cognitive Well-being | 6.52 (2.09) | 7.09 (2.10) |
| Depressive symptoms | 5.63 (4.25) | 5.39 (4.26) |
| Anxiety symptoms | 7.26 (4.69) | 7.37 (4.73) |
| Covid stringency | 62.39 (4.61) | 77.28 (4.19) |

*Note.* Education level refers to primary school (primary), preparatory school for technical and vocational training (low), preparatory school for higher vocational education (middle) and preparatory school for university (high). COVID stringency refers to averaged scores of the daily changing index of COVID containment efforts in the Netherlands.

**Table S8.** Correlation between baseline predictors and compliance in the six weeks cohort

| Measure | 1 | 2 | 3 | 4 | 5 | 6 | 7 |
| --- | --- | --- | --- | --- | --- | --- | --- |
| 1. Compliance (%) | - |  |  |  |  |  |  |
| 2. Age | 0.08 | - |  |  |  |  |  |
| 3. Education | -0.03 | -0.05 | - |  |  |  |  |
| 4. Aff. Well-being | 0.05 | -0.24 | 0.07 | - |  |  |  |
| 5. Cog. Well-being | 0.02 | -0.33 | 0.04 | **0.81** | - |  |  |
| 6. Depression | -0.03 | 0.25 | -0.17 | **-0.71** | **-0.69** | - |  |
| 7. Anxiety | 0.07 | 0.35 | -0.10 | **-0.62** | **-0.63** | **0.73** | - |

*Note.* Bold font indicates significance after implementing FDR correction; Aff. well-being = Affective well-being; Cog. well-being = Cognitive well-being; Adapt. Coping; = Adaptive coping; Maladapt. coping = Maladaptive coping

**Figure S1.** Compliance patterns in the six weeks cohort
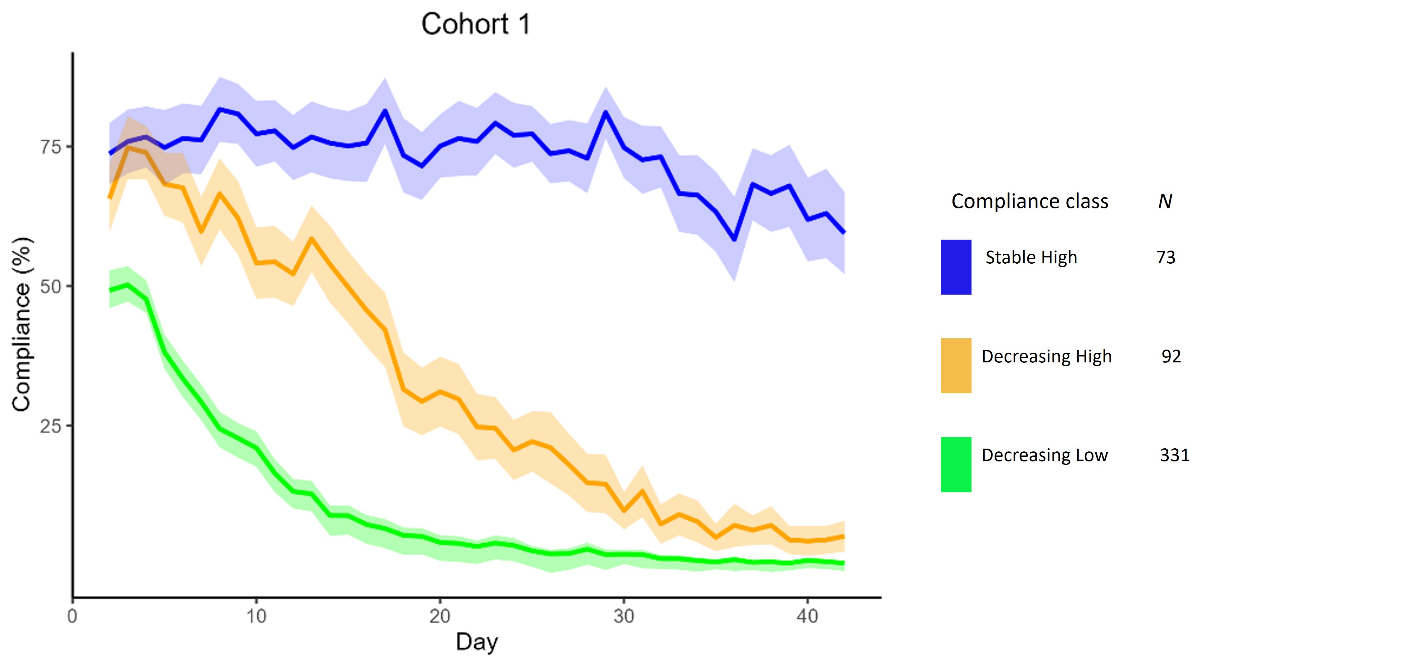


*Note.* Colored lines represent mean compliance levels per day. Shades represent 95% confidence intervals

**Table S9.** Sample distribution by compliance class in the six weeks cohort

| Compliance class | *N* | Intercept (SD) | Slope | *M Compliance* | *M* Age | Gender (%Female) |
| --- | --- | --- | --- | --- | --- | --- |
| Stable high | 73 | 0.69 (0.006) | -0.0004 | 27.8% | 16.9 | 74.9% |
| Decreasing high | 92 | 0.76 (0.005) | -0.0036 | 67.2% | 17.1 | 86.9% |
| Decreasing low | 331 | 0.31 (0.002) | -0.0020 | 10.2% | 15.9 | 74.4% |

**Table S10.** Overview of latent class growth analyses and model selection for the six weeks cohort

|  | loglik | npm | AIC | BIC | entropy | Posterior probabilities | %class1 | %class2 | %class3 | %class4 |
| --- | --- | --- | --- | --- | --- | --- | --- | --- | --- | --- |
| 1-class model | -57327.3 | 3 | 114660 | 114673.3 | 1.00 | - | 100 |  |  |  |
| 2-class model | -41607.8 | 6 | 83227 | 83253 | 0.99 | > 99% | 83.01 | 16.98 |  |  |
| **3-class model** | **-38695.7** | **9** | **77409** | **77447.6** | **0.99** | **>98%** | **19.11** | **14.86** | **66.02** |  |
| 4-class model | -38695.7 | 12 | 77415 | 77466.35 | 0.99 | - | 19.11 | 14.86 | 66.02 | 0 |

*Note.* loglik = Log-Likelihood of model; npm = number of parameters; entropy = degree of class separation from 0 to 1; posterior probability = minimum likelihood of participants to end up in their assigned class; %class1 to %class5 = percentage of participants assigned to that particular class; Bold font indicates the selected model.

**Table S11.** Associations between participant characteristics and compliance classes in the six weeks cohort

| Affective well-being |  | Stable High compliance (vs. Decreasing Low) |  |  | Decreasing High compliance (vs. Decreasing Low) |  |
| --- | --- | --- | --- | --- | --- | --- |
|  | **β** | CI (2.5%; 97.5%) | *p* | **β** | CI (2.5%; 97.5%) | *p* |
| Female gender | 0.00 | -0.25; 0.26 | .987 | -0.39 | -0.68; -0.09 | .011 |
| Age | 0.31 | 0.06; 0.57 | .016 | 0.33 | 0.09; 0.57 | .005 |
| Education | 0.01 | -0.25; 0.27 | .942 | -0.02 | -0.25; 0.20 | .835 |
| Aff. Well-being | 0.19 | -0.09; 0.47 | .185 | 0.11 | -0.14; 0.36 | .393 |
| Covid-stringency | -0.06 | -0.31; 0.19 | .657 | 0.04 | -0.19; 0.28 | .732 |

Cognitive well-being

|  | **β** | CI (2.5%; 97.5%) | *p* | **β** | CI (2.5%; 97.5%) | *p* |
| --- | --- | --- | --- | --- | --- | --- |
| Female gender | 0.02 | -0.24; 0.27 | .910 | -0.37 | -0.67; -0.08 | .014 |
| Age | 0.30 | 0.04; 0.56 | .024 | 0.32 | 0.08; 0.56 | .010 |
| Education | 0.011 | -0.24; 0.27 | .913 | -0.02 | -0.25; 0.21 | .860 |
| Cog. Well-being | 0.09 | -0.19; 0.37 | .529 | 0.01 | -0.24; 0.26 | .926 |
| Covid-stringency | -0.041 | -0.29; 0.21 | .747 | 0.05 | -0.18; 0.29 | .662 |

Depressive symptoms

|  | **β** | CI (2.5%; 97.5%) | *p* | **β** | CI (2.5%; 97.5%) | *p* |
| --- | --- | --- | --- | --- | --- | --- |
| Female gender | -0.00 | -0.26; 0.25 | .974 | -0.38 | -0.67; -0.08 | .013 |
| Age | 0.31 | 0.06; 0.57 | .016 | 0.32 | 0.08; 0.55 | .008 |
| Education | -0.01 | -0.27; 0.25 | .943 | -0.02 | -0.25; 0.21 | .842 |
| Depression | -0.19 | -0.48; 0.09 | .194 | -0.03 | -0.27; 0.22 | .836 |
| Covid stringency | -0.06 | -0.32; 0.19 | .621 | 0.05 | -0.19; 0.29 | .682 |

Anxiety symptoms

|  | **β** | CI (2.5%; 97.5%) | *p* | **β** | CI (2.5%; 97.5%) | *p* |
| --- | --- | --- | --- | --- | --- | --- |
| Gender | 0.04 | -0.22; 0.30 | .774 | -0.40 | -0.69; -0.09 | .009 |
| Age | 0.26 | -0.00; 0.52 | .053 | 0.35 | 0.11; 0.59 | .005 |
| Education | 0.02 | -0.24; 0.27 | .889 | -0.03 | -0.26; 0.20 | .806 |
| Anxiety | 0.05 | -0.23; 0.33 | .733 | -0.12 | -0.38; 0.14 | .353 |
| Covid-stringency | -0.03 | -0.28; 0.22 | .822 | 0.04 | -0.19; 0.28 | .733 |

*Note.* Bold font indicates significance after applying FDR correction

**Figure S2.** Changes in mental health and well-being between compliance classes in the six weeks cohort

**
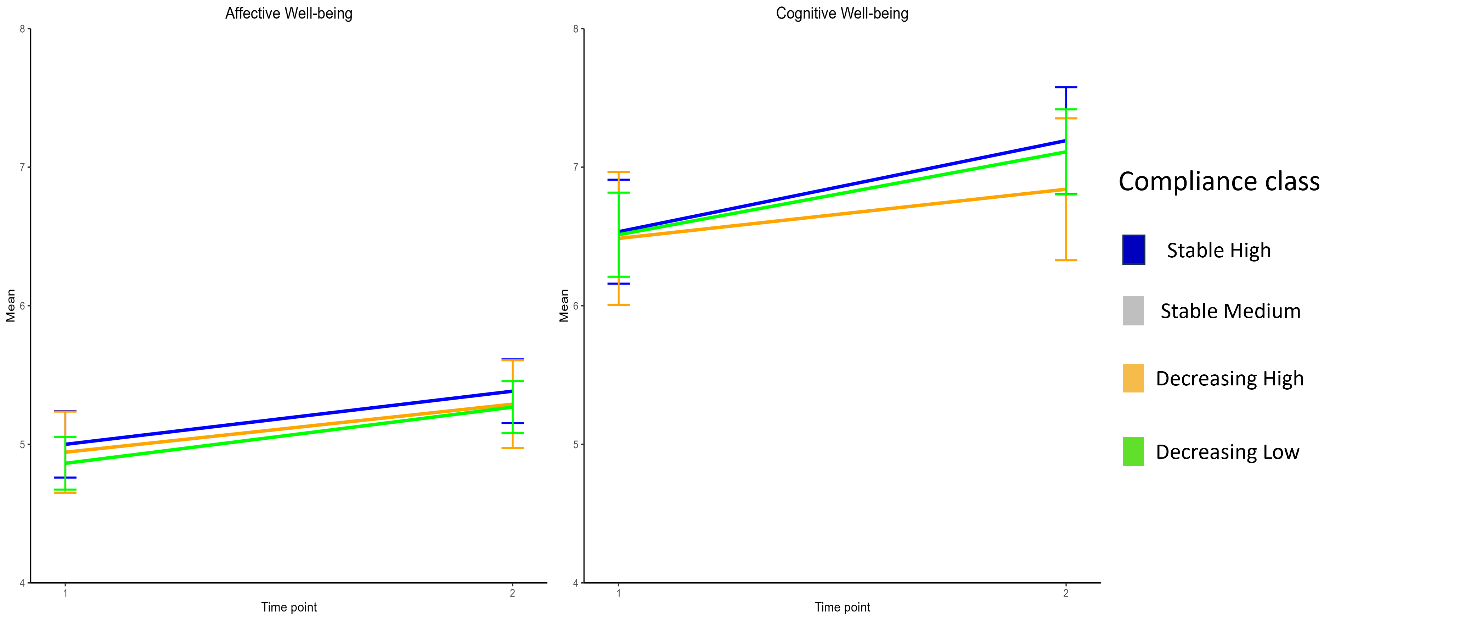
**

**
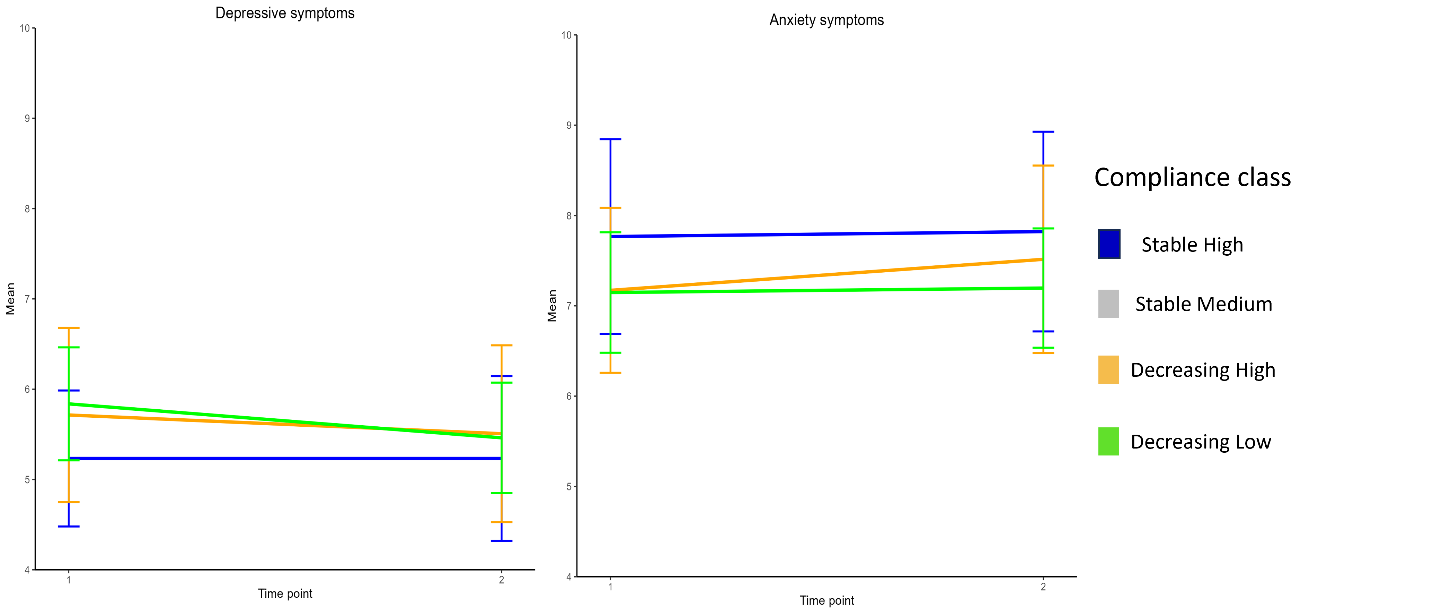
**

*Note.* Lines represent mean levels per class; Whiskers represent 95% confidence intervals

**Table S12.** Fixed effects in multilevel models of changes in well-being, depressive symptoms and anxiety between baseline (T1) and follow-up (T2) in the six weeks cohort.

| Aff. Well-being |  |  | |  | |  | |  | | |  |  |  |  |  |  |  |  |  |  |  |  |  |  |  |  |  |  |
| --- | --- | --- | --- | --- | --- | --- | --- | --- | --- | --- | --- | --- | --- | --- | --- | --- | --- | --- | --- | --- | --- | --- | --- | --- | --- | --- | --- | --- |
|  | Entire cohort | | | | | |  | | Stable High | | | | |  | | Decreasing High | | | |  | | Decreasing Low | | | |  | |  |
|  | B | | *SE* | | *t* | | *p* | | | B | | *SE* | *t* | | *p* | | B | *SE* | *t* | | *p* | | B | *SE* | *t* | | *p* | |
| (Intercept) | 4.98 | | 0.87 | | 5.73 | | <.001 | | | 3.16 | | 1.50 | 2.10 | | .041 | | 6.09 | 1.72 | 3.54 | | <.001 | | 5.56 | 1.30 | 4.27 | | <.001 | |
| Time point | 0.39 | | 0.07 | | 5.55 | | **<.001** | | | 0.36 | | 0.13 | 2.78 | | **.006** | | 0.34 | 0.17 | 1.99 | | .051 | | 0.42 | 0.09 | 4.43 | | **<.001** | |
| Age | -0.07 | | 0.02 | | -3.48 | | **<.001** | | | -0.05 | | 0.04 | -1.44 | | .171 | | -0.06 | 0.04 | -1.51 | | .138 | | -0.09 | 0.03 | -2.95 | | **.003** | |
| Female | 0.37 | | 0.13 | | 2.67 | | **.010** | | | 0.32 | | 0.23 | 1.46 | | .177 | | 0.32 | 0.39 | 0.82 | | .412 | | 0.42 | 0.19 | 2.26 | | .029 | |
| Education | 0.21 | | 0.08 | | 2.73 | | **.007** | | | 0.07 | | 0.13 | 0.53 | | .598 | | 0.45 | 0.17 | 2.61 | | **.013** | | 0.16 | 0.11 | 1.39 | | .171 | |
| COVID stringency | 0.00 | | 0.01 | | 0.15 | | .860 | | | 0.03 | | 0.02 | 1.80 | | .074 | | -0.02 | 0.03 | -0.95 | | .384 | | -0.00 | 0.02 | -0.13 | | .889 | |
| Cog. Well-being |  | |  | |  | |  | | |  | |  |  | |  | |  |  |  | |  | |  |  |  | |  | |
|  | B | | *SE* | | *t* | | *p* | | | B | | *SE* | *t* | | *p* | | B | *SE* | *t* | | *p* | | B | *SE* | *t* | | *p* | |
| (Intercept) | 7.39 | | 1.38 | | 5.36 | | <.001 | | | 2.61 | | 2.25 | 1.16 | | 0.25 | | 9.75 | 2.88 | 3.39 | | .002 | | 8.59 | 2.06 | 4.16 | | <.001 | |
| Time point | 0.57 | | 0.11 | | 5.26 | | **<.001** | | | 0.64 | | 0.22 | 2.90 | | **.005** | | 0.33 | 0.24 | 1.38 | | .173 | | 0.62 | 0.14 | 4.34 | | **<.001** | |
| Age | -0.17 | | 0.03 | | -5.03 | | **<.001** | | | -0.09 | | 0.06 | -1.55 | | .139 | | -0.16 | 0.07 | -2.31 | | .025 | | -0.21 | 0.05 | -4.11 | | **<.001** | |
| Female | 0.78 | | 0.22 | | 3.59 | | **<.001** | | | 0.85 | | 0.34 | 2.52 | | **.019** | | 0.61 | 0.65 | 0.93 | | .351 | | 0.83 | 0.29 | 2.79 | | **.001** | |
| Education | 0.32 | | 0.12 | | 2.56 | | .012 | | | 0.26 | | 0.20 | 1.30 | | .191 | | 0.62 | 0.29 | 2.15 | | .039 | | 0.20 | 0.18 | 1.12 | | .275 | |
| COVID stringency | 0.00 | | 0.02 | | 0.06 | | .947 | | | 0.06 | | 0.03 | 2.05 | | .050 | | -0.04 | 0.04 | -1.00 | | .356 | | -0.01 | 0.03 | -0.18 | | .843 | |
| Depressive Symptoms |  | |  | |  | |  | | |  | |  |  | |  | |  |  |  | |  | |  |  |  | |  | |
|  | B | | *SE* | | *t* | | *p* | | | B | | *SE* | *t* | | *p* | | B | *SE* | *t* | | *p* | | B | *SE* | *t* | | *p* | |
| (Intercept) | 11.67 | | 3.08 | | 3.79 | | <.001 | | | 16.88 | | 5.93 | 2.85 | | .010 | | 8.23 | 6.41 | 1.28 | | .202 | | 10.05 | 4.46 | 2.25 | | .028 | |
| Time point | -0.26 | | 0.15 | | -1.74 | | .095 | | | 0.01 | | 0.27 | 0.18 | | .999 | | -0.25 | 0.25 | -1.00 | | .322 | | -0.39 | 0.23 | -1.73 | | .119 | |
| Age | 0.14 | | 0.07 | | 1.94 | | .052 | | | 0.06 | | 0.15 | 0.39 | | .696 | | 0.01 | 0.15 | 0.08 | | .937 | | 0.25 | 0.11 | 2.25 | | .025 | |
| Female | -1.74 | | 0.49 | | -3.58 | | **<.001** | | | -1.81 | | 0.89 | -2.03 | | .047 | | -2.25 | 1.45 | -1.55 | | .126 | | -1.73 | 0.64 | -2.69 | | .009 | |
| Education | -1.31 | | 0.28 | | -4.72 | | **<.001** | | | -0.92 | | 0.53 | -1.76 | | .084 | | -1.72 | 0.64 | -2.69 | | **.009** | | -1.19 | 0.39 | -3.07 | | **.003** | |
| COVID stringency | -0.05 | | 0.04 | | -1.09 | | .282 | | | -0.13 | | 0.08 | -1.70 | | .097 | | 0.07 | 0.09 | 0.68 | | .508 | | -0.05 | 0.06 | -0.78 | | .447 | |
| Anxiety Symptoms |  | |  | |  | |  | | |  | |  |  | |  | |  |  |  | |  | |  |  |  | |  | |
|  | B | | *SE* | | *t* | | *P* | | | B | | *SE* | *t* | | *P* | | B | *SE* | *t* | | *p* | | B | *SE* | *t* | | *p* | |
| (Intercept) | 10.01 | | 3.22 | | 3.11 | | .002 | | | 14.01 | | 7.19 | 1.95 | | .051 | | 8.28 | 6.02 | 1.37 | | .176 | | 8.21 | 4.56 | 1.80 | | .078 | |
| Time point | 0.11 | | 0.20 | | 0.57 | | .643 | | | 0.08 | | 0.37 | 0.23 | | .885 | | 0.42 | 0.39 | 1.07 | | .430 | | 0.01 | 0.29 | 0.05 | | .913 | |
| Age | 0.37 | | 0.08 | | 4.85 | | **<.001** | | | 0.34 | | 0.18 | 1.91 | | .069 | | 0.30 | 0.14 | 2.13 | | .038 | | 0.41 | 0.11 | 3.69 | | **<.001** | |
| Female | -2.76 | | 0.51 | | -5.42 | | **<.001** | | | -4.38 | | 1.08 | -4.05 | | **<.001** | | -2.54 | 1.36 | -1.87 | | .067 | | -2.29 | 0.66 | -3.51 | | **<.001** | |
| Education | -0.57 | | 0.29 | | -1.96 | | .055 | | | 0.44 | | 0.64 | 0.69 | | .465 | | -0.89 | 0.59 | -1.49 | | .138 | | -0.82 | 0.39 | -2.05 | | .043 | |
| COVID stringency | -0.06 | | 0.04 | | -1.43 | | .156 | | | -0.12 | | 0.09 | -1.32 | | .185 | | -0.02 | 0.09 | -0.23 | | .831 | | -0.04 | 0.06 | -0.68 | | .506 | |

Note. Bold font indicates coefficients with significance *p* <.0041. Aff. Well-being = Affective Well-being; Cog. Well-being = Cognitive Well-being; We used the values of cognitive and affective well-being, depression, and anxiety at T1 to predict the respective values at T2. Coefficients indicate the significance of change in the variable from T1 to T2 when controlled for the other variables.

**Table S13.** Pairwise comparisons between compliance classes in developments of well-being, depression, and anxiety in the six weeks cohort

| Affective well-being | |  |  |  |  |  | Cognitive well-being | | |  | |  | |  | |  |
| --- | --- | --- | --- | --- | --- | --- | --- | --- | --- | --- | --- | --- | --- | --- | --- | --- |
| Time point | Class 1 | Class 2 | n1 | n2 | *p* |  | | Time point | Class 1 | | Class 2 | | n1 | | n2 | *p* |
| 1 | St. High | Decr. High | 73 | 70 | .790 |  | 1 | | St. High | | Decr. High | | 73 | | 70 | .888 |
| 1 | St. High | Decr. Low | 73 | 197 | .436 |  | 1 | | St. High | | Decr. Low | | 73 | | 197 | .939 |
| 1 | Decr. High | Decr. Low | 70 | 197 | .655 |  | 1 | | Decr. High | | Decr. Low | | 70 | | 197 | .925 |
| 2 | St. High | Decr. High | 73 | 70 | .665 |  | 2 | | St. High | | Decr. High | | 73 | | 70 | .321 |
| 2 | St. High | Decr. Low | 73 | 197 | .516 |  | 2 | | St. High | | Decr. Low | | 73 | | 197 | .779 |
| 2 | Decr. High | Decr. Low | 70 | 197 | .906 |  | 2 | | Decr. High | | Decr. Low | | 70 | | 197 | .362 |
| Depression |  |  |  |  |  |  | Anxiety | |  | |  | |  | |  |  |
| Time point | Class 1 | Class 2 | n1 | n2 | *p* |  | Time point | | Class 1 | | Class 2 | | n1 | | n2 | *p* |
| 1 | St. High | Decr. High | 73 | 70 | .494 |  | 1 | | St. High | | Decr. High | | 73 | | 70 | .442 |
| 1 | St. High | Decr. Low | 73 | 197 | .294 |  | 1 | | St. High | | Decr. Low | | 73 | | 197 | .329 |
| 1 | Decr. High | Decr. Low | 70 | 197 | .833 |  | 1 | | Decr. High | | Decr. Low | | 70 | | 197 | .970 |
| 2 | St. High | Decr. High | 73 | 70 | .703 |  | 2 | | St. High | | Decr. High | | 73 | | 70 | .700 |
| 2 | St. High | Decr. Low | 73 | 197 | .700 |  | 2 | | St. High | | Decr. Low | | 73 | | 197 | .342 |
| 2 | Decr. High | Decr. Low | 70 | 197 | .940 |  | 2 | | Decr. High | | Decr. Low | | 70 | | 197 | .637 |

*Note.* St. High = Stable High; St. Med. = Stable Medium; Decr. High = Decreasing High; Decr. Low = Decreasing Low
